# EXO1 overexpression induces homologous recombination deficiency and enhances PARP inhibitor sensitivity in ER-positive breast cancer: modulation by N4BP2L2-Mediated restoration

**DOI:** 10.3389/fcell.2025.1695627

**Published:** 2025-11-14

**Authors:** Runa Sugiyama, Anna S. Sedukhina, Eri Sato, Ayako Yamaura, Kimino Minagawa, Sookhee Pae, Ena Imai, Ankita Chawla, Ziran Xu, Mihika Chakraborty, Satori Gonoi, Jotaro Yamaoka, Kazuo Yudo, Koichiro Tsugawa, Ko Sato

**Affiliations:** 1 Department of Breast and Endocrine Surgery, Graduate School of Medicine, St. Marianna University, Kawasaki, Japan; 2 Department of Frontier Medicine, Institute of Medical Science, Graduate School of Medicine, St. Marianna University, Kawasaki, Japan; 3 Shirokane Sanko Clinic Research Centre, Minato, Japan; 4 Division of Genomic Epidemiology and Clinical Trials, Clinical Trials Research Center, Nihon University School of Medicine, Itabashi, Japan; 5 K International School Tokyo, Koto, Japan; 6 St. Mary’s International School, Setagaya, Japan

**Keywords:** Exo1, homologous recombination, breast cancer, N4BP2L2, PARP inhibitor, therapeutic response

## Abstract

Exonuclease 1 (EXO1) is a critical enzyme in homologous recombination (HR) and is implicated in cancer progression, with overexpression linked to poor prognosis in multiple tumor types. Yet, the impact of EXO1 overexpression on HR efficiency in estrogen receptor (ER)-positive breast cancer remains unclear. Here, we investigated this using The Cancer Genome Atlas (TCGA) and functional studies in ER-positive T47D cells. High EXO1 expression was associated with elevated homologous recombination deficiency (HRD) scores in ER-positive tumors, indicating impaired HR activity. In T47D cells, EXO1 overexpression reduced HR efficiency, measured by the Advanced Homologous Recombination Assay (ASHRA), and increased sensitivity to the PARP inhibitor olaparib. Using multi-cohort transcriptomic analysis and machine learning interpretability approaches (Random Forest, SHAP, and permutation importance), we identified N4BP2L2 as a key modulator of HR under EXO1 overexpression. Both SHAP and permutation-importance analyses consistently highlighted N4BP2L2 as a strong HR-restorative gene, whereas OTUD7B showed weaker, context-dependent effects. Validation in an independent Korean cohort confirmed N4BP2L2 as a reproducible modulator of HR. Survival analyses across three ER-positive breast cancer cohorts (TCGA, E-MTAB-365, and METABRIC) revealed that high EXO1 expression was associated with shorter survival, whereas concurrent high N4BP2L2 expression mitigated this adverse prognostic effect, even after multivariate adjustment. Functional assays in both T47D and MCF7 cells demonstrated that co-expression of N4BP2L2 restored HR activity and reduced olaparib sensitivity in EXO1-overexpressing cells. These findings suggest EXO1 overexpression serves as a marker of functional HR deficiency and a potential predictor of PARP inhibitor response, highlighting the EXO1–N4BP2L2 axis as a promising biomarker and therapeutic target, especially for guiding PARP inhibitor use beyond BRCA-mutated tumors.

## 1 Introduction

Breast cancer is a molecularly diverse disease, with its intrinsic subtypes classified based on gene expression profiles into basal-like, luminal A, luminal B, HER2-enriched, and normal-like groups (Perou et al., 2000). These subtypes exhibit distinct biological behaviors and therapeutic responses, highlighting the necessity of subtype-specific treatment strategies (Sorlie et al., 2001; Parker et al., 2009; Prat and Perou, 2011). The PAM50 assay, a widely used tool for breast cancer classification, assesses the expression of 50 genes to stratify tumors into these intrinsic subtypes and predict recurrence risk (Nielsen et al., 2010). Basal-like breast cancer, often triple-negative, is frequently associated with high genomic instability and homologous recombination (HR) deficiency, which typically results from BRCA1 mutations or dysfunctions (Lord and Ashworth, 2016). On the other hand, estrogen receptor (ER)-positive breast cancer, primarily comprising luminal subtypes, is generally thought to maintain proficient DNA repair mechanisms, with HR deficiency being a less common feature (Feng et al., 2023).

To investigate the broader role of EXO1 expression across these subtypes, we began our analysis by leveraging the PAM50 data. While EXO1 overexpression has predominantly been associated with basal-like breast cancers, our analysis of the PAM50 data revealed that EXO1 is also elevated in a subset of ER-positive tumors, suggesting that EXO1’s involvement in HR deficiency might extend beyond the basal-like subtype. This observation warranted further exploration of EXO1’s potential impact on HR function in ER-positive breast cancers, which are traditionally considered HR-proficient.

HR is a critical DNA repair pathway that resolves double-strand breaks (DSBs) by using a homologous DNA template, thus ensuring high-fidelity repair (Jasin and Rothstein, 2013). Central to HR is BRCA1, which facilitates DNA end resection by displacing the 53BP1 complex and recruiting the MRE11–CtIP complex (Symington and Gautier, 2011; Chapman et al., 2013). This process is further extended by Exonuclease 1 (EXO1), an exonuclease that generates single-stranded DNA (ssDNA) tracts (Nimonkar et al., 2011). These ssDNA regions are initially coated by replication protein A (RPA), which is then replaced by RAD51 to initiate strand invasion and repair (San Filippo et al., 2008; Heyer et al., 2010). Disruption of this pathway—either through genetic mutations or altered regulatory mechanisms—can compromise HR efficiency, contributing to tumorigenesis and influencing therapeutic responses (Lord and Ashworth, 2012; Ceccaldi et al., 2015).

The clinical success of poly (ADP-ribose) polymerase (PARP) inhibitors, which exploit HR deficiency for synthetic lethality, has revolutionized the treatment of BRCA-mutant cancers (Bryant et al., 2005; Farmer et al., 2005). While this strategy is well established for BRCA1-deficient tumors, its broader applicability across other cancers requires a deeper understanding of HR regulation beyond classical gene mutations (Lord and Ashworth, 2017). For example, in preclinical models, the loss of 53BP1 has been shown to restore HR activity in BRCA1-deficient cells, potentially affecting PARP inhibitor sensitivity—though this phenomenon remains rare in clinical tumors (Bouwman et al., 2010; Jaspers et al., 2013).

To further understand the complex regulatory network governing HR, it is essential to explore genetic interactions that modulate HR efficiency. Machine learning approaches, when applied to large-scale genomic datasets, can uncover subtle patterns and combinatorial effects that traditional methods might overlook, offering new insights into the regulation of DNA repair pathways and their implications for treatment (Fabris et al., 2017).

EXO1, a key player in DNA end resection, has been shown to be overexpressed in several tumor types, including liver and lung cancers (Liu et al., 2025). In these cancers, elevated EXO1 expression is associated with poor survival outcomes (Dai et al., 2018; Zhou et al., 2021). However, the precise contribution of EXO1 overexpression to HR efficiency remains unclear. Because excessive EXO1 activity has the potential to disrupt the delicate balance of DNA repair, its overexpression may represent an underexplored mechanism of HR dysregulation. In this study, we examined the impact of EXO1 overexpression on HR in ER-positive breast cancer. Using bioinformatics analysis of The Cancer Genome Atlas (TCGA), *in vitro* assays with the ER-positive T47D cell line, and machine learning approaches to identify genetic modifiers of HR, we demonstrate that EXO1 overexpression exerts a previously unrecognized suppressive effect on HR. Furthermore, we identified candidate genes that modulate this effect and experimentally validated their roles in shaping HR proficiency and therapeutic response. In addition, we extended our validation to an independent ER-positive cell model (MCF7) and performed multivariate survival analyses across multiple cohorts, further strengthening the mechanistic and clinical significance of our findings. Together, these findings provide new insights into the regulation of HR in breast cancer and suggest potential avenues for improving treatment strategies in ER-positive disease.

## 2 Materials and methods

### 2.1 Data acquisition and preprocessing

Transcriptomic and clinical data for Invasive Breast Carcinoma (TCGA, GDC) were obtained from cBioPortal on 27 January 2025. Two transcriptomic datasets were downloaded: TPM expression values (data_mRNA_seq_tpm.txt) and raw read counts (data_mRNA_seq_read_counts.txt). Gene identifiers were mapped to gene symbols using the Entrez_GENE_symbol.txt file. TPM data were restricted to ER-positive breast cancer patients based on IHC status in clinical data and normalized using Z-score transformation. Z-score normalization was applied gene-wise across samples after TPM normalization, such that each gene’s expression distribution was centered to a mean of 0 and a standard deviation of one across the cohort. This approach allows direct comparison of relative gene expression across patients and ensures appropriate scaling for downstream machine learning–based HRD classification. Homologous recombination deficiency (HRD) scores were obtained from a previous study and matched to patients (Zhang et al., 2021). Among ER-positive patients, those with EXO1 expression Z-score >0 were stratified into HRD-high (HRD ≥42, Group 2) and HRD-low (HRD <42, Group 1) groups.

To assess reproducibility of machine learning-identified genes from TCGA, an independent dataset of ER-positive breast cancers from a multi-omics study of younger Asian patients was utilized (Kan et al., 2018). HRD scores in this dataset were calculated using *expHRD*, an individualized transcriptome-based prediction model for homologous recombination deficiency assessment in cancer, to validate gene reproducibility.

### 2.2 Differentially expressed gene analysis

Differential gene expression analysis between HRD-high (G2, HRD ≥42) and HRD-low (G1, HRD <42) groups was performed using raw read count data derived from RNA-seq experiments. Three widely used statistical frameworks were applied: DESeq2, edgeR, and EBSeq, each of which is designed for count-based transcriptomic data analysis but differs in its underlying statistical assumptions and modeling strategies.

DESeq2 models count data using the negative binomial distribution and incorporates shrinkage estimators for dispersion and fold changes, improving statistical power and interpretability. edgeR also employs a negative binomial model but utilizes empirical Bayes methods to estimate gene-wise dispersions, which enhances performance, particularly in datasets with small sample sizes. EBSeq uses an empirical Bayesian hierarchical model to identify differentially expressed genes by modeling expression states as mixtures across groups.

For downstream analysis, we retained only genes that were identified as significantly differentially expressed by both DESeq2 and edgeR, using a false discovery rate (FDR) threshold of 0.001. In both tools, FDR correction was performed using the Benjamini–Hochberg (BH) procedure.

### 2.3 Machine learning modeling

Four machine learning algorithms—Random Forest, XGBoost, LightGBM, and Support Vector Machines—were trained on the 1,405 common differentially expressed genes to classify HRD status among ER-positive patients with EXO1 Z-score >0. This aimed to identify genes predictive of HRD. All models were trained using 5-fold stratified cross-validation to ensure balanced representation of HRD-high and HRD-low groups across folds. Class imbalance was mitigated by balanced subsampling within each iteration. Model performance was evaluated by the mean area under the receiver operating characteristic curve (AUC). Feature importance for the Random Forest model was ranked using both Gini importance and permutation importance, and interpretability was further examined by SHAP (Shapley Additive exPlanations) analysis to quantify the contribution and directionality of each gene to HRD prediction. All analyses were performed in Python (v3.10) using scikit-learn (v1.3) and SHAP (v0.42).

### 2.4 Cell culture and transfection

T47D (ATCC HTB-133™) and MCF7 (ATCC HTB-22™) human breast cancer cell lines were obtained from the American Type Culture Collection (ATCC). T47D cells were cultured in RPMI-1640 medium supplemented with 10% fetal bovine serum (FBS), and MCF7 cells were maintained in ATCC-formulated Eagle’s Minimum Essential Medium (EMEM) containing 10% FBS, following the vendor’s protocols. Both cell lines were incubated at 37 °C in a humidified atmosphere of 5% CO_2_ and passaged at a 1:3–1:6 ratio, with medium renewal two to three times per week. Transient transfection was performed using PEI-MAX (Polysciences) at a PEI:DNA ratio of 3:1 (w/w) with 6 µg total DNA per 10-cm dish. Plasmids included pPB-Neo-CAG > ORF-Stuffer (vector control; VB900131-3591nmk), pPB-Neo-CAG-hEXO1 (VB220411-1024uvw), pPB-Neo-CAG-N4BP2L2 (VB250323-1300uvw), and the EXO1 + N4BP2L2 co-expression construct (VectorBuilder). For the Advanced Homologous Recombination Assay (ASHRA), cells were co-transfected with donor plasmid pBS-ACTB-2000-GFP-fr1 (Addgene) and Cas9/guide RNA plasmid (LentiCRISPRv2-ACTB-C1, Addgene #169796) at a 2:1 mass ratio. LentiCRISPRv2-scramble (Addgene #169795) served as a non-targeting control. Cells were harvested 48 h post-transfection for RNA extraction, HR assays, and colony formation analysis.

### 2.5 RNA isolation and cDNA synthesis

Total RNA was extracted from cultured cells (≤5 × 10^6^) using the ReliaPrep™ RNA Cell Miniprep System (Promega) with on-column DNase I digestion. RNA was eluted in 30 µL nuclease-free water and stored at −80 °C. cDNA was synthesized from 500 ng RNA using PrimeScript RT Master Mix (Takara) in a 10 µL reaction following the manufacturer’s protocol.

### 2.6 Quantitative real-time PCR (qPCR)

qPCR was performed using SYBR® Green reagents (Thermo Fisher) on a StepOne™ Real-Time PCR System (Applied Biosystems). Reactions (10 µL) included cDNA template, primers, and master mix. Cycling: 95 °C 10 min; 40 cycles of 95 °C 15 s and 60 °C 1 min; followed by melt curve analysis. Triplicate technical and biological replicates were performed. Relative expression was calculated by the ΔΔCt method, normalized to GAPDH.

### 2.7 Primers

N4BP2L2: F 5′-CAGACAGGTTTGTGAACCAGCAG-3, R 5′-GCCATCACGATTCTGACCAAGC-3′

EXO1: F 5′-GCAACTTCTTCGTGAGGG A-3′, R 5-AGGAAGGTATTGTTGGCCCG-3′

GAPDH: F 5′-GGTGAAGGTCGGTGTGAACG-3′ R 5′-CTCGCTCCTGGAAGATGGTG-3′

### 2.8 Homologous recombination (HR) assay

HR efficiency was determined as previously described (Pae et al., 2024). GFP transcript levels from the knock-in allele were normalized to the control allele using triplicate biological and technical replicates. NTCs were included to verify assay specificity.

### 2.9 HR primers

Target Forward Reverse.

Knock-in allele GTCCTGCTGGAGTTCGTGACCG GTGCAATCAAAGTCCTCGGC.

Control allele AGTTGCGTTACACCCTTTCTTG GTGCAATCAAAGTCCTCGGC.

### 2.10 Colony formation assay with olaparib

The Colony Formation assay was performed as previously described (Nagasawa et al., 2015; Nakagawa et al., 2015). Briefly, 48 hours after transfection, cells were reseeded in 6-well plates at 500–1,000 cells/well and treated with Olaparib at concentrations of 0, 0.01, 0.1, 1, 10, and 100 µM. After 7 days, colonies were fixed, stained with crystal violet, washed, and air-dried. Experimental groups included EXO1 overexpression, N4BP2L2 overexpression, co-expression of EXO1 + N4BP2L2, and vector control. Ct values were exported from the instrument software, and relative quantification (ΔΔCt) was computed as described. Colony counts obtained from ImageQuant were used to calculate relative survival across Olaparib doses, with plating efficiency correction where applicable. Statistical analyses and graphing were performed in Prism or equivalent software as indicated in figure legends.

### 2.11 Survival and Multivariate Cox regression analyses

Disease-free and overall survival analyses were performed using the TCGA, E-MTAB-365, and METABRIC cohorts. Survival curves were estimated by the Kaplan-Meier method and compared using the log-rank test. Multivariate Cox proportional hazards models were fitted with age and clinical stage as covariates. For E-MTAB-365, Scarff–Bloom–Richardson grade was substituted for stage due to dataset limitations. All analyses were conducted in R (v4.3.2) using the “survival” and “survminer” packages.

### 2.12 Statistical analysis

Statistical significance was assessed using two-tailed t-tests for comparisons between two groups. For all analyses, a p-value of <0.05 was considered statistically significant. Error bars represent the standard error of the mean (SEM) from at least three independent experiments unless otherwise specified.

## 3 Results

### 3.1 EXO1 expression across breast cancer subtypes and its functional impact on homologous recombination

**FIGURE 1:**
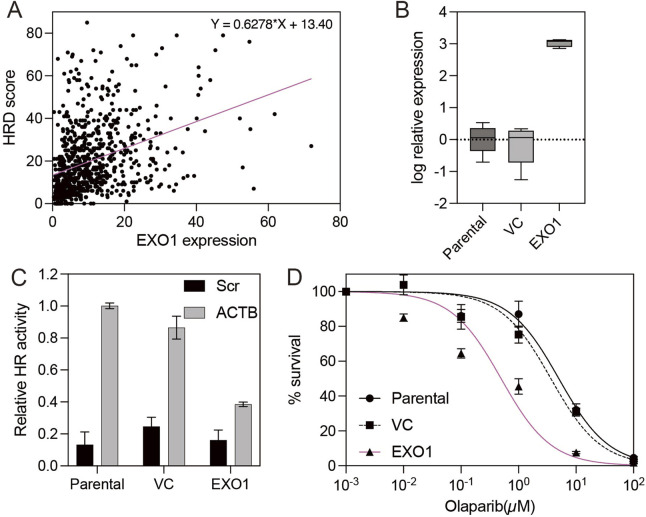
EXO1 overexpression induces homologous recombination deficiency and sensitizes ER-positive breast cancer cells to PARP inhibition **(A)** Correlation between EXO1 mRNA expression and homologous recombination deficiency (HRD) scores in ER-positive breast cancers from the TCGA dataset. The red line indicates the linear regression fit. **(B)** The box plot shows EXO1 expression measured by real-time RT-PCR in parental T47D cells, vector control-transfected cells (VC), and EXO1-overexpressing T47D cells (EXO1). Error bars represent the minimum and maximum values. **(C)** Homologous recombination (HR) efficiency, assessed by β-actin-GFP transcript levels using RT-PCR, in parental T47D cells, vector control-transfected cells (VC), and EXO1-overexpressing cells (EXO1). Error bars represent the standard error of the mean (SEM) from four independent experiments. **(D)** Sensitivity to the PARP inhibitor olaparib in parental T47D cells, vector control-transfected cells (VC), and EXO1-overexpressing cells (EXO1). Cell viability was measured after drug treatment. Error bars represent the standard error of the mean (SEM) from three independent experiments.

Analysis of The Cancer Genome Atlas (TCGA) dataset revealed that EXO1 is highly expressed in basal-like breast cancer according to the PAM50 classification (Supplementary Figure S1). While EXO1 is a component gene of the PAM50 panel and is characteristically overexpressed in basal-like tumors, elevated expression was also observed in a subset of estrogen receptor (ER)-positive breast cancers, indicating a broader role for EXO1 beyond basal-like tumors (Supplementary Figure S1) (Nielsen et al., 2010). Given that homologous recombination (HR) deficiency is commonly associated with BRCA1 loss and basal-like breast cancer, we hypothesized that ER-positive breast cancer—where BRCA1 is typically intact—would provide a valuable model to study the effect of EXO1 overexpression on HR (Lord and Ashworth, 2016; Feng et al., 2023). To investigate this, we examined the relationship between EXO1 expression and HR efficiency. HR efficiency was inferred using the HRD (homologous recombination deficiency) score, a composite genomic metric integrating loss of heterozygosity (LOH), telomeric allelic imbalance (TAI), and large-scale state transitions (LST) (Telli et al., 2016). Notably, we observed a significant positive correlation between EXO1 expression and HRD scores in ER-positive breast cancer (Figure 1A), suggesting that high EXO1 levels are associated with impaired HR function in this subtype. To validate the functional consequences of EXO1 overexpression on HR, we used the ER-positive breast cancer cell line T47D. HR activity was quantified using the Advanced Homologous Recombination Assay (ASHRA), a CRISPR/Cas9-based system that measures HR by tracking integration of a GFP sequence into the endogenous ACTB locus (Yoshino et al., 2019). Unlike the traditional DR-GFP assay, ASHRA provides a more reliable correlation between HR activity and cellular sensitivity to genotoxic stress (Pierce et al., 1999; Yoshino et al., 2019). We compared HR activity among parental T47D cells, vector control (VC) cells, and EXO1-overexpressing cells. Both parental and VC cells successfully integrated GFP into the ACTB locus following ACTB-specific gRNA targeting, producing a β-actin-GFP fusion transcript (Figures 1B,C). In contrast, EXO1-overexpressing cells failed to produce this fusion transcript regardless of the gRNA used, indicating suppressed HR activity (Figures 1B,C). Given the known link between HR deficiency and sensitivity to PARP inhibitors, we further tested whether EXO1 overexpression sensitized T47D cells to PARP inhibitor olaparib (McCabe et al., 2006). As expected, EXO1-overexpressing cells exhibited significantly increased sensitivity to olaparib compared to parental and VC cells (Figures 1B,D), supporting the notion that EXO1 overexpression induces functional HR deficiency and enhances therapeutic vulnerability.

### 3.2 Identification of genes modulating homologous recombination in EXO1-overexpressing ER-positive breast cancer

**FIGURE 2:**
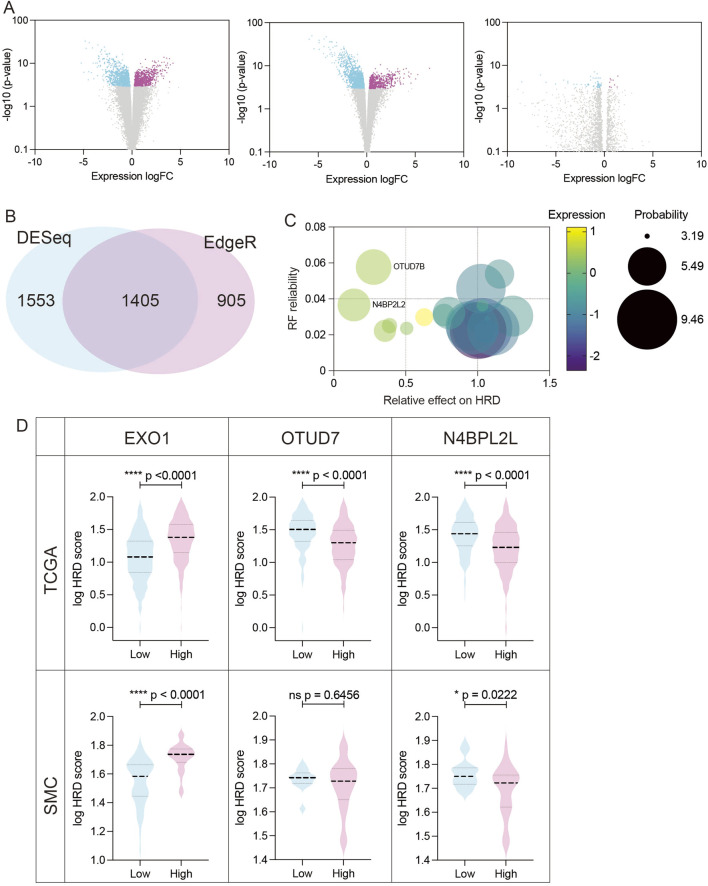
Identification and validation of candidate genes modulating HR efficiency in EXO1-overexpressing ER-positive breast cancer **(A)** Volcano plots showing differentially expressed genes (DEGs) between high-HRD (HRD score ≥42) and low-HRD (HRD score <42) groups in EXO1-overexpressing ER-positive breast cancers, analyzed using DESeq2 (left), edgeR (middle), and EBSeq (right). **(B)** Venn diagram illustrating the overlap of DEGs identified by DESeq2 and edgeR. A total of 1,405 overlapping genes were selected for downstream analysis. **(C)** Feature importance ranking of candidate genes based on the Random Forest model. N4BP2L2 and OTUD7B were identified as top modulators of HRD, with their relative impact on HRD scores shown. **(D)** Validation of candidate gene associations with HRD scores in both the TCGA and Korean breast cancer cohort (SMC dataset). Boxplots show HRD scores in EXO1-overexpressing tumors stratified by high vs. low expression of EXO1, OTUD7B, and N4BP2L2. Statistical significance was assessed using two-tailed t-tests.

BRCA1 dysfunction is a well-established cause of homologous recombination deficiency (HRD), yet cells with concurrent loss of both BRCA1 and 53BP1 can paradoxically regain HR proficiency (Bouwman et al., 2010; Jaspers et al., 2013). Although the co-occurrence of BRCA1 and 53BP1 dysfunction in clinical tumors remains rare and poorly characterized, understanding this compensatory mechanism is vital due to its implications for resistance to PARP inhibitors and treatment strategies (Bouwman et al., 2010). In this study, we demonstrated that EXO1 overexpression induces HR deficiency and sensitizes ER-positive breast cancer cells to PARP inhibition, highlighting its relevance in therapeutic stratification. To further elucidate the genetic landscape underlying HR modulation in EXO1-overexpressing ER-positive breast cancers, we aimed to identify gene expression profiles associated with both HR efficiency and PARP inhibitor sensitivity. Using the TCGA breast cancer dataset (n = 282 EXO1-overexpressing ER-positive cases, ∼20,000 genes per sample), we stratified tumors into high- and low-HRD score groups based on a widely used cutoff value of 42, which has been established as a clinically relevant threshold for defining HR deficiency (Choi and Lee, 2022). We then conducted differential expression analysis between these two groups. To minimize potential biases due to class imbalance, we employed three established methods: DESeq2, edgeR, and EBSeq (Soneson and Delorenzi, 2013; Etoh and Nakao, 2023). Comparison of results revealed that DESeq2 and edgeR produced consistent and biologically plausible volcano plot distributions, while EBSeq exhibited an irregular pattern—particularly in the non-significant gene range—likely due to its optimization for small-sample analyses (Barber et al., 2013) (Figure 2A). Given the large sample size and gene set, we excluded EBSeq from further analysis. We identified 1,405 differentially expressed genes (DEGs) common to both DESeq2 and edgeR for downstream investigation (Figure 2B).

Next, we applied four machine learning models—Hist Gradient Boosting Classifier, XGBoost, Support Vector Machine (SVM), and Random Forest—to identify genes most predictive of HRD status in the EXO1-overexpressing subset (Fumagalli et al., 2023; Chen and Kabir, 2024; Das et al., 2024; Ghuriani et al., 2025; Sharda et al., 2025). Among these, the Random Forest model achieved the highest performance, as measured by area under the curve (AUC) (Table 1), and was selected for further analysis. This model revealed a subset of genes whose elevated expression either increased or decreased HRD scores (Figure 2C). Notably, while the genes associated with elevated HRD scores exerted relatively modest effects (up to 1.3-fold increase), those linked to reduced HRD had a more pronounced impact. We focused on two top candidates—N4BP2L2 and OTUD7B—whose overexpression substantially lowered HRD scores, suggesting restoration of HR function (Figure 2C). To validate these findings, we examined the Korean breast cancer cohort (SMC dataset) (Kan et al., 2018). While OTUD7B expression did not correlate with HRD in this independent dataset, N4BP2L2 overexpression consistently reduced HRD scores, confirming its potential as a robust modulator of HR (Figure 2D). Together, these findings position N4BP2L2 as a key genetic suppressor of EXO1-induced HR deficiency, with potential implications for developing strategies to reverse HRD and modulate therapeutic response in ER-positive breast cancers.

**TABLE 1:** Performance of machine learning models.

Model name	AUC	Accuracy (%)	PR-AUC	MCC
Random Forest	0.949	0.872	0.823	0.471
XG Boost	0.887	0.865	0.633	0.441
SVM	0.931	0.865	0.794	0.431
HGBC	0.865	0.854	0.484	0.411

Abbreviations: SVM, support vector machine; HGBC, hist gradient boosting classifier.

### 3.3 Model interpretability identifies HR-restorative genes

**FIGURE 3:**
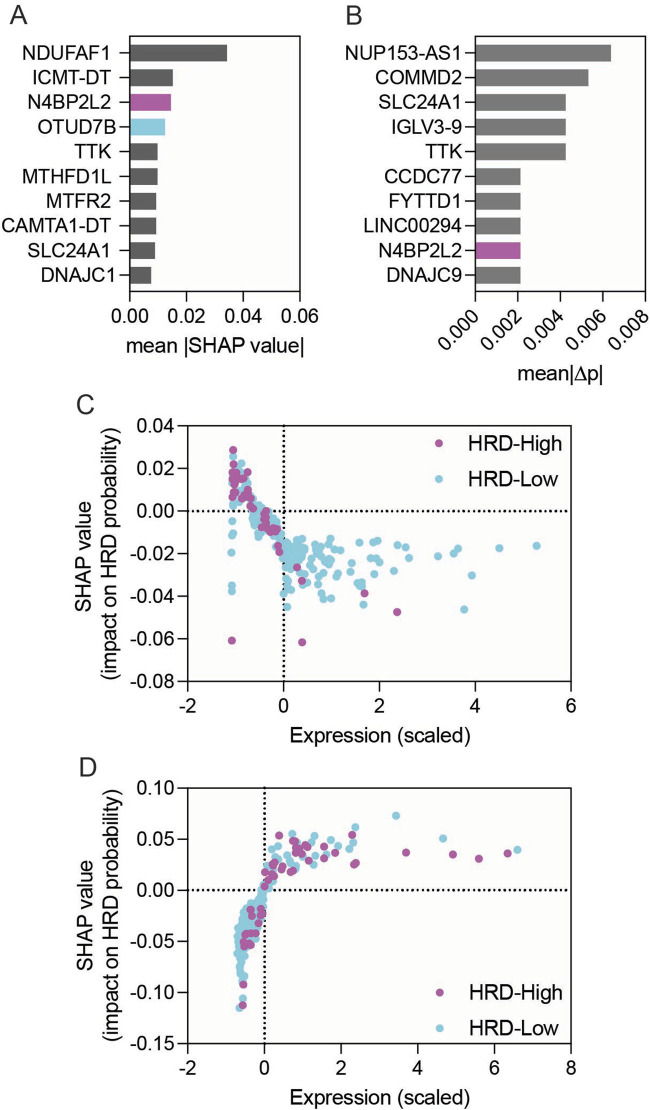
Model interpretability analysis of genes associated with HRD status in EXO1-overexpressing ER-positive breast cancer. **(A)** Bar graph showing the top 10 genes ranked by mean |SHAP| values derived from the Random Forest model. **(B)** Bar graph showing permutation-importance scores for the same top 10 genes. **(C,D)** SHAP dependence plots for N4BP2L2 **(C)** and OTUD7B **(D)**, showing relationships between scaled gene-expression values and SHAP impact on predicted HRD probability.

To better interpret the Random Forest model and visualize how individual gene expression levels influenced HRD prediction, we incorporated SHAP (Shapley Additive exPlanations) and permutation-importance analyses (Figure 3). Both approaches provided complementary interpretability to the Random Forest classifier. The SHAP summary plot ranked genes by their mean absolute SHAP values (Figure 3A; Supplementary Figure S2). Both N4BP2L2 and OTUD7B were within the highest-ranked features, supporting their potential involvement in HR modulation. Permutation-importance analysis independently confirmed N4BP2L2 as one of the top contributors, whereas the relative impact of OTUD7B was modest (Figure 3B; Supplementary Figure S3). SHAP dependence plots further revealed that higher N4BP2L2 expression was consistently associated with reduced predicted HRD probability, supporting its HR-restorative role (Figure 3C). In contrast, OTUD7B showed variable directionality in its SHAP profile, suggesting a context-dependent effect (Figure 3D). These findings align with the lack of reproducibility of OTUD7B’s HRD-lowering association in the SMC validation cohort (Figure 2D). Together, the combined DEG, machine-learning, and model-interpretability analyses position N4BP2L2 as a key genetic suppressor of EXO1-induced HR deficiency, providing both statistical and mechanistic evidence for its role in restoring HR function and influencing therapeutic response in ER-positive breast cancers.

To provide clinical context for the survival analyses, baseline characteristics of patients in the TCGA, E-MTAB-365, and METABRIC cohorts were summarized (Supplementary Table S1). The SMC cohort was excluded because survival data were not available. In the METABRIC cohort, EXO1-high tumors exhibited a significantly higher stage distribution compared with EXO1-low tumors (p = 0.001), whereas this difference was attenuated when N4BP2L2 was co-expressed (p = 0.587). These findings suggest that N4BP2L2 may counteract the clinically aggressive features associated with EXO1 overexpression. Recurrence status and follow-up duration were summarized in Supplementary Table S1 to provide context for relapse-free survival analyses. The E-MTAB-365 dataset lacked stage information but included the Scarff–Bloom–Richardson grade, which was used as a surrogate histologic variable.

Survival analyses were then performed across three independent ER-positive breast cancer cohorts—TCGA, E-MTAB-365, and METABRIC (Supplementary Figure S4). TCGA represents an RNA-seq–based dataset, whereas E-MTAB-365 and METABRIC are microarray-based, together covering distinct technological platforms and patient populations. In TCGA, no significant association between gene expression and disease-free survival was observed (log-rank p = 0.7477). In contrast, in the E-MTAB-365 dataset, high EXO1 expression was associated with inferior survival (HR = 1.860, 95% CI 1.196–2.893; p = 0.0076), whereas N4BP2L2 alone showed no effect (p = 0.769). Importantly, the adverse prognostic impact of EXO1 was attenuated in patients with concurrent high N4BP2L2 expression (HR = 1.210, 95% CI 0.6758–2.166; p = 0.4938). This pattern was reproduced in the larger METABRIC dataset, where EXO1-high cases exhibited significantly shorter survival (HR = 1.766, 95% CI 1.455–2.143; p < 0.0001), while dual EXO1 + N4BP2L2 overexpression mitigated this effect (HR = 1.056, 95% CI 0.575–1.278; p = 0.575).

Multivariate Cox regression models adjusted for age and stage confirmed these results across both E-MTAB-365 and METABRIC (Supplementary Table S2). In METABRIC, high EXO1 expression remained an independent predictor of shorter survival (HR = 1.37, 95% CI 1.10–1.70; p = 0.0042), whereas in E-MTAB-365 the association was borderline (HR = 1.67, 95% CI 1.00–2.77; p = 0.0500). Notably, even after multivariate adjustment, the hazard associated with EXO1 overexpression was markedly diminished in the dual EXO1 + N4BP2L2 high-expression group in both cohorts, supporting that the protective influence of N4BP2L2 is independent of clinical covariates and reflects a true biological mitigation of EXO1-induced aggressiveness.

### 3.4 Functional interplay between EXO1 and N4BP2L2 in ER-positive breast cancer cells

**FIGURE 4:**
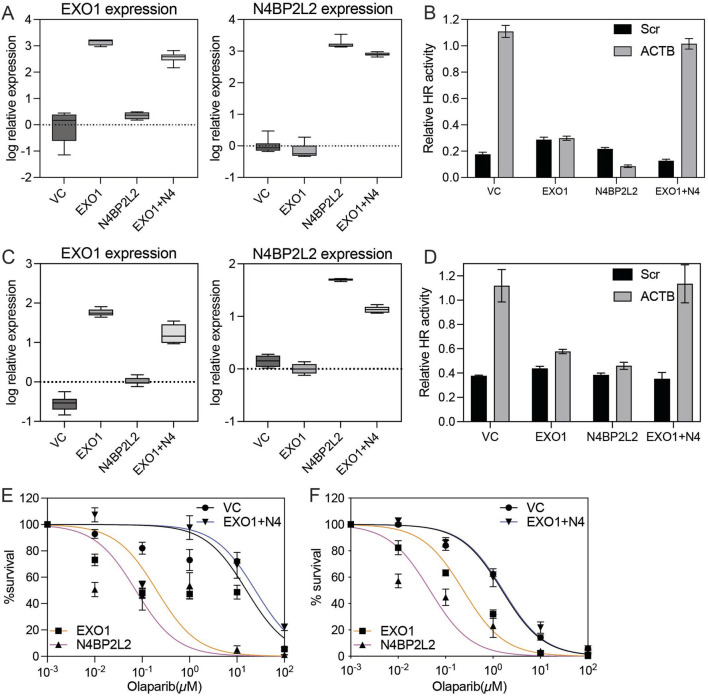
Functional impact of N4BP2L2 on HR efficiency and PARP inhibitor sensitivity in EXO1-overexpressing ER-positive breast cancer cells **(A)** Box plots show EXO1 (left) and N4BP2L2 (right) expression measured by real-time RT-PCR in vector control-transfected (VC), EXO1-overexpressing (EXO1), N4BP2L2-overexpressing (N4BP2L2) and EXO1 + N4BP2L2 co-expressing (EXO1 + N4) T47D cells. Error bars indicate minimum and maximum values. **(B)** Homologous recombination (HR) efficiency, assessed by β-actin-GFP transcript levels using RT-PCR in the same T47D cell groups. Error bars represent the standard error of the mean (SEM) from four independent experiments. “EXO1+N4” denotes co-expression of EXO1 and N4BP2L2. **(C)** Box plots showing EXO1 (left) and N4BP2L2 (right) mRNA expression levels measured by real-time RT-PCR in vector control (VC), EXO1-overexpressing (EXO1), N4BP2L2-overexpressing (N4BP2L2) and EXO1 + N4BP2L2 co-expressing (EXO1 + N4) MCF7 cells. Error bars indicate minimum and maximum values. **(D)** HR efficiency in the same MCF7 groups, assessed by β-actin–GFP transcript levels using ASHRA. Error bars represent the SEM from three independent experiments. **(E,F)** Colony formation assay assessing sensitivity to the PARP inhibitor olaparib in T47D **(E)** and MCF7 **(F)** cells. Cell viability (%) was measured after 7 days of drug exposure. Error bars represent the standard error of the mean (SEM) from three independent experiments.

To functionally validate the role of N4BP2L2 in modulating homologous recombination (HR), we performed assays in ER-positive T47D cells. HR activity was assessed using the Advanced Homologous Recombination Assay (ASHRA), which measures GFP integration at the ACTB locus via CRISPR/Cas9. As expected, vector control (VC) cells showed robust GFP integration, while EXO1-overexpressing cells failed to produce the β-actin–GFP fusion transcript, confirming suppressed HR activity (Figures 4A,B). Strikingly, co-overexpression of N4BP2L2 with EXO1 restored GFP integration, indicating rescue of HR proficiency (Figures 4A,B). We extended these analyses to include MCF7 cells, another ER-positive breast cancer model, to assess whether the EXO1–N4BP2L2 interaction is cell-line specific. In MCF7 cells, EXO1 overexpression similarly suppressed HR activity, whereas co-overexpression of N4BP2L2 restored GFP integration to near-baseline levels (Figures 4C,D). Consistent with these HR assay results, EXO1-overexpressing cells were hypersensitive to the PARP inhibitor olaparib, and this effect was reversed by N4BP2L2 co-expression, restoring resistance to levels comparable with VC cells in both T47D and MCF7 lines (Figures 4E,F). Together with Random Forest analysis and validation in an independent breast cancer cohort, these results identify N4BP2L2 as a robust suppressor of EXO1-induced HR deficiency. These findings demonstrate that the HR-restorative effect of N4BP2L2 is reproducible across multiple ER-positive breast cancer backgrounds, reinforcing its role in counteracting EXO1-induced HR deficiency. This functional interplay highlights the EXO1–N4BP2L2 axis as a potential target for modulating HR capacity and therapeutic response in ER-positive breast cancer.

## 4 Discussion

In this study, we identified EXO1 overexpression as a key modulator of homologous recombination deficiency and a potential predictor of PARP inhibitor sensitivity in ER-positive breast cancer. Through an integrated approach combining bioinformatics analysis, functional validation, and machine learning, we provide compelling evidence that EXO1 overexpression can impair HR and sensitizes ER-positive breast cancer cells to therapeutic intervention with PARP inhibitors. These findings offer novel insights into the molecular mechanisms that contribute to HRD in breast cancer and highlight the translational potential of EXO1 as a biomarker for personalized treatment strategies.

Our analysis of The Cancer Genome Atlas (TCGA) dataset revealed a significant correlation between EXO1 overexpression and elevated HRD scores in ER-positive breast cancer, a subtype that is typically associated with proficient HR repair mechanisms (Feng et al., 2023). This finding contrasts with the established understanding of basal-like breast cancers, which are more commonly linked to defective HR due to BRCA1 mutations (Lord and Ashworth, 2016). Elevated EXO1 expression may therefore represent an alternative mechanism driving HRD in ER-positive tumors. Although EXO1 normally promotes DNA-end resection, its overexpression may lead to uncontrolled (“hyper-“) resection, reduced RPA recycling efficiency and replication-fork instability, collectively impairing RAD51 loading and functional HR capacity (Zhao et al., 2020). These results suggest that EXO1 expression could complement BRCA1/2 genotyping as a functional biomarker for HRD, expanding therapeutic eligibility for PARP-inhibitor–based regimens beyond BRCA-mutated cases.

We further demonstrated the functional consequences of EXO1 overexpression using the ER-positive T47D cell line. Employing the ASHRA, we showed that EXO1 overexpression significantly impairs HR activity, corroborating the bioinformatics data. This impairment of HR activity was accompanied by increased sensitivity to the PARP inhibitor Olaparib, a widely used therapeutic in HR-deficient cancers. Importantly, we extended these analyses to a second ER-positive cell line, MCF7, which reproduced the same pattern: EXO1 overexpression impaired HR and co-expression of N4BP2L2 restored HR proficiency. These results support the concept of EXO1-driven HRD serving as a predictive biomarker for PARP inhibitor sensitivity in ER-positive breast cancer, with potential clinical implications for treatment selection. Collectively, these data demonstrate that the EXO1–N4BP2L2 axis is a reproducible regulator of HR capacity across multiple ER-positive contexts.

To gain deeper insights into the genetic factors influencing HR in EXO1-overexpressing tumors, we applied machine learning to identify potential modulators of HR efficiency. Among the 1,405 DEGs identified, N4BP2L2 and OTUD7B emerged as significant candidates. To interpret the Random-Forest classifier, we applied SHAP (SHapley Additive exPlanations) and permutation-importance analyses. These complementary approaches consistently identified N4BP2L2 as one of the most influential features predicting HRD probability, while OTUD7B exhibited weaker and inconsistent contributions across datasets. SHAP dependence plots indicated that high N4BP2L2 expression lowers predicted HRD probability, supporting its HR-restorative role, whereas OTUD7B’s context-dependent effects likely reflect background-specific regulatory interactions. Consistent with this, validation in the independent SMC cohort confirmed the reproducibility of N4BP2L2, but not OTUD7B, as an HR-suppressive gene. Functional assays further confirmed these computational findings: in EXO1-overexpressing T47D and MCF7 cells, co-expression of N4BP2L2 restored β-actin–GFP integration and normalized HR activity, while also reversing olaparib hypersensitivity. This highlights the complexity of HR regulation in cancer and the necessity of further investigation into the broader genetic landscape of HR modulation. Mechanistically, little is currently known about the biochemical function of N4BP2L2 or its molecular partners. While it shares sequence similarity with NEDD4-binding proteins (Salipante et al., 2009), its precise role remains uncharacterized, and no direct interactions with EXO1 or other homologous recombination factors have been reported. Accordingly, we interpret its HR-restorative function as likely indirect, possibly mediated through regulatory stabilization or modulation of HR-associated processes that warrant future investigation.

To evaluate the clinical relevance of these findings, we analyzed disease-free survival across three independent ER-positive cohorts, TCGA, E-MTAB-365, and METABRIC. Within ER-positive breast cancer, high EXO1 expression correlated with shorter survival in the E-MTAB-365 and METABRIC cohorts, whereas N4BP2L2 alone showed no prognostic impact. Notably, patients with concurrent high EXO1 and N4BP2L2 expression no longer exhibited poor outcomes, indicating that N4BP2L2 expression mitigates the adverse prognostic effect of EXO1 overexpression. This interaction remained significant in multivariate Cox models adjusted for age and stage, underscoring the translational relevance of the EXO1–N4BP2L2 axis in patient prognosis.

While our study provides significant advances in understanding HR regulation in breast cancer, several limitations warrant consideration. First, although our bioinformatics analyses and machine learning models were validated across large, independent datasets, the functional roles of other candidate genes identified in these analyses remain unexplored. Second, mechanistic experiments such as RAD51 foci formation or EXO1–N4BP2L2 interaction assays will be necessary to delineate the precise molecular pathways involved. Third, although the T47D and MCF7 cell lines provided valuable insights, additional *in vivo* validation, such as patient-derived xenografts or organoids, will be required to confirm the translational relevance of our findings. Finally, the clinical utility of EXO1 and N4BP2L2 as biomarkers or therapeutic targets will require validation in clinical trials, which will be essential for determining their potential for patient stratification and treatment personalization.

In conclusion, our study establishes EXO1 overexpression as a key driver of HRD in ER-positive breast cancer and identifies it as a potential predictive marker for PARP inhibitor sensitivity. The discovery of N4BP2L2 as a modulator capable of restoring HR proficiency and mitigating the poor clinical outcomes associated with EXO1 hyperactivity expands the biological and translational relevance of our findings. Integrating machine learning, functional assays, and survival analyses, this study provides mechanistic and clinical insight into the EXO1–N4BP2L2 axis and its role in shaping HRD-related therapeutic vulnerabilities. These findings enhance our understanding of HR regulation in breast cancer and pave the way for the development of personalized treatment strategies aimed at exploiting HRD-related vulnerabilities. Moving forward, further exploration of HR modulators and their clinical applications will be critical for optimizing therapeutic outcomes in breast cancer patients.

## Data Availability

The datasets presented in this study can be found in online repositories. The names of the repository/repositories and accession number(s) can be found in the article/Supplementary Material, further inquiries can be directed to the corresponding author.
