# Supplementary material for: EXO1 overexpression induces homologous recombination deficiency and enhances PARP inhibitor sensitivity in ER-positive breast cancer: modulation by N4BP2L2-Mediated restoration
Source: Front Cell Dev Biol. 2025 Nov 14;13:1695627. doi: 10.3389/fcell.2025.1695627 (PMC12660296; doi:10.3389/fcell.2025.1695627)
Supplement: Supplementary file 1 [file DataSheet2.pdf]

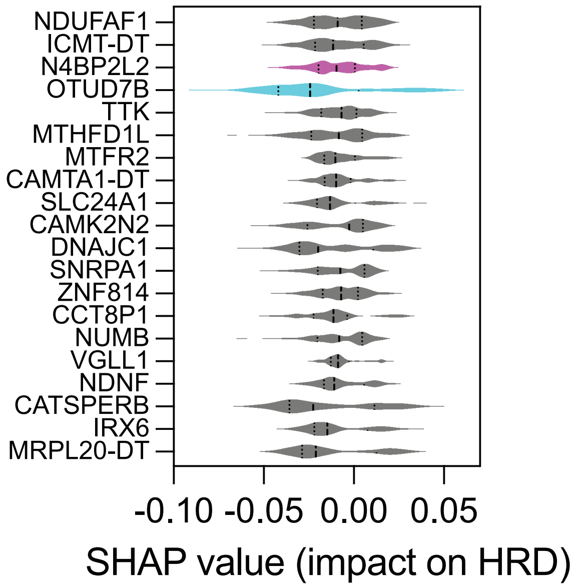

Supplementary Figure 2

SHAP beeswarm plot summarizing top 20 genes ranked by mean |SHAP| value. The plot summarizes the top 20 genes contributing to HRD prediction based on SHAP (Shapley Additive exPlanations) values from the Random Forest model trained on 1405 differentially expressed genes. Each dot represents an individual patient sample, plotted according to the gene's SHAP value, which reflects both the magnitude and direction of its influence on the predicted HRD probability. Higher absolute SHAP values indicate stronger impact on model output. N4BP2L2 and OTUD7B rank among the most influential features, supporting their roles as potential modulators of homologous recombination status.
