# Supplementary material for: EXO1 overexpression induces homologous recombination deficiency and enhances PARP inhibitor sensitivity in ER-positive breast cancer: modulation by N4BP2L2-Mediated restoration
Source: Front Cell Dev Biol. 2025 Nov 14;13:1695627. doi: 10.3389/fcell.2025.1695627 (PMC12660296; doi:10.3389/fcell.2025.1695627)
Supplement: Supplementary file 2 [file DataSheet4.pdf]

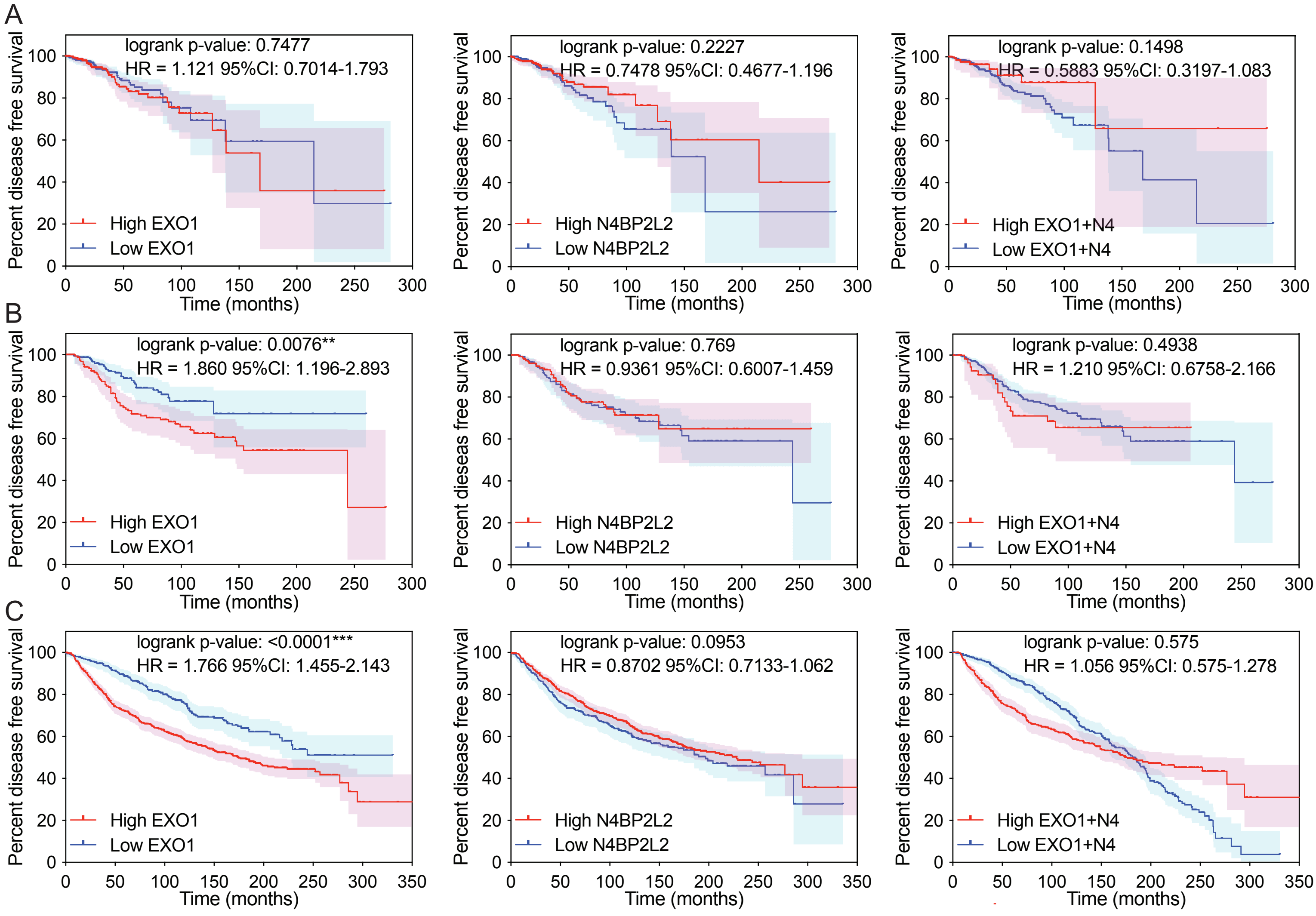

Supplementary Figure 4.
Survival analysis of EXO1 and N4BP2L2 expression across three independent ER-positive breast cancer cohorts.
Kaplan–Meier curves show disease-free survival according to expression levels of EXO1, N4BP2L2, and combined EXO1 + N4BP2L2 status in (A) TCGA, (B) E-MTAB-365, and (C) METABRIC datasets.
For each cohort, patients were dichotomized by the median expression of the indicated genes. Log-rank p-values and hazard ratios (HR) with 95% confidence intervals are displayed within each panel. Tick marks denote censored observations. Analyses were limited to ER-positive cases with available clinical follow-up.
