# Supplementary material for: EXO1 overexpression induces homologous recombination deficiency and enhances PARP inhibitor sensitivity in ER-positive breast cancer: modulation by N4BP2L2-Mediated restoration
Source: Front Cell Dev Biol. 2025 Nov 14;13:1695627. doi: 10.3389/fcell.2025.1695627 (PMC12660296; doi:10.3389/fcell.2025.1695627)
Supplement: Supplementary file 3 [file DataSheet6.pdf]

TCGA: EXO1

	Crude			Adjusted		
	HR	95%C.I.	p-value	HR	95%C.I.	p-value
Expression	1.140	0.70-1.85	0.6002	1.170	0.72-1.90	0.5387
Stage	2.000	1.41-2.83	0.0001***	2.040	1.45-2.88	<0.0001***
Age	1.020	1.00-1.04	0.1095	1.00	1.00-1.04	0.0452*

TCGA: N4BP2L2

	Crude			Adjusted		
	HR	95%C.I.	p-value	HR	95%C.I.	p-value
Expression	1.140	0.70-1.85	0.6002	1.170	0.47-1.25	0.5387
Stage	2.000	1.41-2.83	0.0001***	2.040	1.45-2.88	<0.0001***
Age	1.020	1.00-1.04	0.1095	1.00	1.00-1.04	0.0452*

TCGA: EXO1/N4BP2L2

	Crude			Adjusted		
	HR	95%C.I.	p-value	HR	95%C.I.	p-value
Expression	1.140	0.70-1.85	0.6002	1.170	0.30-1.31	0.5387
Stage	2.000	1.41-2.83	0.0001***	2.040	1.45-2.88	<0.0001***
Age	1.020	1.00-1.04	0.1095	1.00	1.00-1.04	0.0452*

M-META-365: EXO1

	Crude			Adjusted		
	HR	95%C.I.	p-value	HR	95%C.I.	p-value
Expression	1.970	1.21-3.20	0.0062**	1.670	1.00-2.77	0.0500
Stage	1.710	1.16-2.53	0.0071**	1.840	1.22-2.77	0.0380*
Age	1.010	0.99-1.03	0.1882	1.020	1.00-1.04	0.0977

M-META-365: N4BP2L2

	Crude			Adjusted		
	HR	95%C.I.	p-value	HR	95%C.I.	p-value
Expression	0.950	0.60-1.49	0.8161	1.120	0.70-1.79	0.6283
Stage	1.710	1.16-2.53	0.0071**	1.840	1.22-2.77	0.0034*
Age	1.010	0.99-1.03	0.1882	1.020	1.00-1.04	0.0795

M-META-365: EXO1/N4BP2L2

	Crude			Adjusted		
	HR	95%C.I.	p-value	HR	95%C.I.	p-value
Expression	1.410	0.82-2.41	0.2166	1.340	0.77-2.33	0.2998
Stage	1.710	1.16-2.53	0.0071**	1.760	1.17-2.65	0.0067*
Age	1.010	0.99-1.03	0.1882	1.020	1.00-1.04	0.0714

METABRIC: EXO1

	Crude			Adjusted		
	HR	95%C.I.	p-value	HR	95%C.I.	p-value

Expression	1.440	1.16-1.78	0.0009***	1.370	1.10-1.70	0.0042*
Stage	1.700	1.42-2.03	<0.0001***	1.680	1.40-2.02	<0.0001***
Age	1.000	0.99-1.01	0.3916	1.000	0.99-1.01	0.5892

METABRIC: N4BP2L2

	Crude HR	95%C.I.	p-value	Adjusted HR	95%C.I.	p-value
Expression	0.950	0.77-1.18	0.6543	1.010	0.81-1.25	0.9363
Stage	1.700	1.42-2.03	<0.0001***	1.720	1.43-2.06	<0.0001***
Age	1.000	0.99-1.01	0.3916	1.000	0.99-1.01	0.7066

METABRIC: EXO1/N4BP2L2

	Crude HR	95%C.I.	p-value	Adjusted HR	95%C.I.	p-value
Expression	1.240	0.96-1.60	0.1034	1.220	0.95-1.58	0.1231
Stage	1.700	1.42-2.03	<0.0001***	1.720	1.43-2.06	<0.0001***
Age	1.000	0.99-1.01	0.3916	1.000	0.99-1.01	0.6376

Supplementary Table 2.
Multivariate Cox regression analyses evaluating disease-free survival in ER-positive breast cancer cohorts.
The TCGA and METABRIC models were adjusted for age and pathological stage.
The E-METAB-365 dataset lacks stage information; therefore, models were adjusted for age and histological grade (Scarff-Bloom-Richardson).
Hazard ratios (HR) and 95% confidence intervals (CI) are shown for high versus low expression of EXO1, N4BP2L2, and their combined expression groups.
$p < 0.05$ was considered statistically significant.
