# Supplementary material for: EXO1 overexpression induces homologous recombination deficiency and enhances PARP inhibitor sensitivity in ER-positive breast cancer: modulation by N4BP2L2-Mediated restoration
Source: Front Cell Dev Biol. 2025 Nov 14;13:1695627. doi: 10.3389/fcell.2025.1695627 (PMC12660296; doi:10.3389/fcell.2025.1695627)
Supplement: Supplementary file 4 [file DataSheet3.pdf]

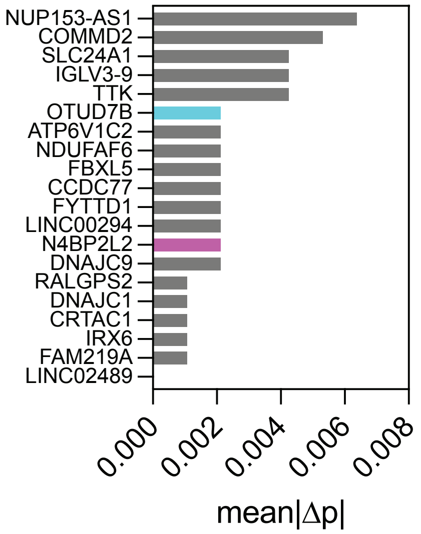

Supplementary Figure 3

Permutation importance ranking of the top 20 genes

Bars represent mean |Δp| values, indicating average change in predicted HRD probability upon feature permutation.
