# Supplementary material for: EXO1 overexpression induces homologous recombination deficiency and enhances PARP inhibitor sensitivity in ER-positive breast cancer: modulation by N4BP2L2-Mediated restoration
Source: Front Cell Dev Biol. 2025 Nov 14;13:1695627. doi: 10.3389/fcell.2025.1695627 (PMC12660296; doi:10.3389/fcell.2025.1695627)
Supplement: Supplementary file 5 [file DataSheet1.pdf]

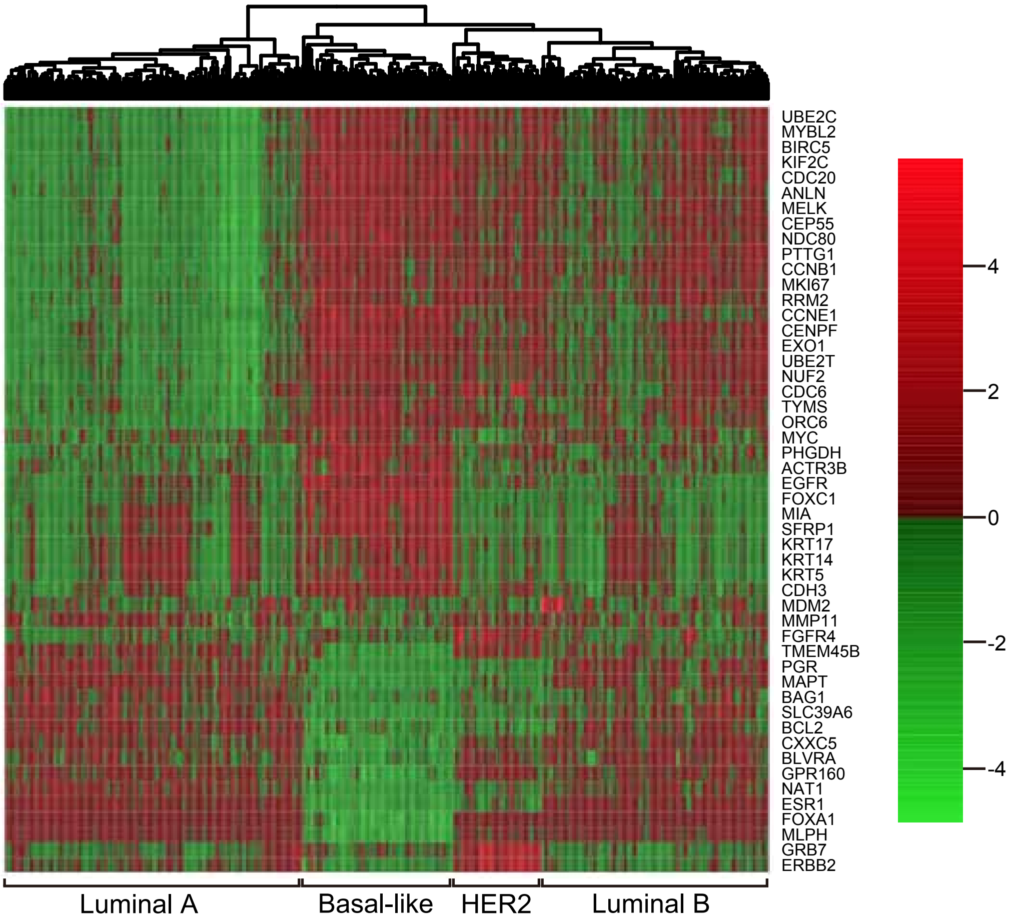

Supplementary Figure 1.

EXO1 expression across PAM50 intrinsic subtypes in the TCGA breast cancer cohort

mRNA expression levels of EXO1 across the PAM50-defined intrinsic subtypes in the TCGA breast cancer cohort.
