# Supplementary material for: EXO1 overexpression induces homologous recombination deficiency and enhances PARP inhibitor sensitivity in ER-positive breast cancer: modulation by N4BP2L2-Mediated restoration
Source: Front Cell Dev Biol. 2025 Nov 14;13:1695627. doi: 10.3389/fcell.2025.1695627 (PMC12660296; doi:10.3389/fcell.2025.1695627)
Supplement: Supplementary file 6 [file DataSheet5.pdf]

TCGA		EXO1			N4BP2L2			EXO1+N4BP2L2		
		High	Low	p-value	High	Low	p-value	High	Low	p-value
Total		363	360		362	361		133	590	
Age	Mean (SD)	58.2 (13.33)	59.12 (12.58)	0.335	58.45 (12.17)	58.86 (13.72)	0.670	58.65 (12.29)	58.66 (13.12)	0.997
Stage	I (SD)	50 (13.8)	77 (21.4)	0.062	63 (17.4)	64 (17.7)	0.861	17 (12.8)	110 (18.6)	0.194
	II (SD)	216 (59.5)	198 (55.0)		211 (58.3)	203 (56.2)		83 (62.4)	331 (56.1)	
	III (SD)	93 (25.6)	81 (22.5)		85 (23.5)	89 (24.7)		33 (24.8)	141 (23.9)	
	IV (SD)	4 (1.1)	4 (1.1)		3 (0.8)	5 (1.4)		0 (0.0)	8 (1.4)	
RFS month	Mean (SD)	36.03 (35.18)	39.52(35.12)	0.183	39.47 (37.09)	36.06 (33.09)	0.192	37.21 (41.06)	37.89 (33.74)	0.841
Recurrence	n (%)	33 (9.1)	32 (8.9)	1.000	31 (8.6)	34 (9.4)	0.786	8 (6.0)	57 (9.7)	0.246

M-MTAB-365		EXO1			N4BP2L2			EXO1+N4BP2L2		
		High	Low	p-value	High	Low	p-value	High	Low	p-value
Total		154	147		149	152		56	245	
Age	Mean (SD)	56.27 (13.30)	57.69 (11.64)	0.328	56.64 (12.15)	57.28 (12.89)	0.662	55.43 (13.24)	57.31 (12.34)	0.310
Grade	1 (SD)	4 (2.6)	28 (19.0)	<0.001	19 (12.8)	13 (8.6)	0.03	2 (3.6)	30 (12.2)	0.04
	2 (SD)	93 (60.4)	100 (68.0)		102 (68.5)	91 (59.9)		34 (60.7)	159 (64.9)	
	3 (SD)	57 (37.0)	19 (12.9)		28 (18.8)	48 (31.6)		20 (35.7)	56 (22.9)	
RFS month	Mean (SD)	83.43 (59.10)	74.43 (45.38)	0.102	68.86 (47.47)	88.04 (56.46)	0.002	72.18 (54.37)	80.00 (52.68)	0.320
Recurrence	n (%)	53 (34.4)	24 (16.3)	0.001	33 (22.1)	44 (28.9)	0.223	17 (30.4)	60 (24.5)	0.320

METABRIC		EXO1			N4BP2L2			EXO1+N4BP2L2		
		High	Low	p-value	High	Low	p-value	High	Low	p-value
Total		402	422		407	417		165	659	
Age	Mean (SD)	63.12 (12.14)	62.08 (12.61)	0.232	63.72 (11.81)	61.49 (12.86)	0.01	64.35 (10.95)	62.15 (12.7)	0.041
Stage	I (SD)	120 (29.9)	165 (39.2)	0.001	156 (38.4)	129 (30.9)	0.03	50 (30.3)	235 (35.7)	0.587
	II (SD)	241 (60.0)	239 (56.8)		230 (56.7)	250 (60.0)		103 (62.4)	377 (57.3)	
	III (SD)	36 (9.0)	12 (3.6)		18 (4.4)	33 (7.9)		11 (6.7)	40 (6.1)	
	IV (SD)	5 (1.2)	2 (0.5)		2 (0.5)	5 (1.2)		1 (0.6)	6 (0.9)	
RFS month	Mean (SD)	108.29 (78.38)	119.03 (70.93)	0.039	117.69 (75.8)	109.99 (73.73)	0.14	107.11 (77.42)	115.46 (74.10)	0.200
Recurrence	n (%)	188 (46.8)	151 (35.8)	0.002	168 (41.3)	171 (41.0)	0.994	76 (46.1)	263 (39.9)	0.178

Supplementary Table 1.

Baseline clinicopathologic characteristics of ER-positive breast-cancer cohorts used for survival analyses

Clinicopathologic variables for TCGA, E-MTAB-365, and METABRIC cohorts stratified by EXO1-high, N4BP2L2-high, and dual EXO1 + N4BP2L2-high expression groups.

Values are shown as counts (%) for categorical variables and mean ± standard deviation (SD) for continuous variables.

p-values were calculated using Student's *t*-test for continuous and Fisher's exact tests for categorical comparisons.

Stage information was unavailable in E-MTAB-365; the Scarff–Bloom–Richardson grade was used as a histologic comparator.

These data provide the baseline context for the multivariate Cox regression models presented in Supplementary Table S2.
